# Cytokine Production but Lack of Proliferation in Peripheral Blood Mononuclear Cells from Chronic Chagas' Disease Cardiomyopathy Patients in Response to *T. cruzi* Ribosomal P Proteins

**DOI:** 10.1371/journal.pntd.0002906

**Published:** 2014-06-05

**Authors:** Silvia A. Longhi, Augusto Atienza, Graciela Perez Prados, Alcinette Buying, Virginia Balouz, Carlos A. Buscaglia, Radleigh Santos, Laura M. Tasso, Ricardo Bonato, Pablo Chiale, Clemencia Pinilla, Valeria A. Judkowski, Karina A. Gómez

**Affiliations:** 1 Instituto de Investigaciones en Ingeniería Genética y Biología Molecular (INGEBI) - Consejo Nacional de Investigaciones Científicas y Tecnológicas (CONICET), Buenos Aires, Argentina; 2 Facultad de Farmacia y Bioquímica, Universidad de Buenos Aires, Buenos Aires, Argentina; 3 Hospital General de Agudos J.M. Ramos Mejía, Buenos Aires, Argentina; 4 Hospital General de Agudos J. A. Fernández, Buenos Aires, Argentina; 5 Torrey Pines Institute for Molecular Studies, San Diego, California, United States of America; 6 Instituto de Investigaciones Biotecnológicas “Dr. Rodolfo Ugalde”, Universidad Nacional de San Martín (UNSAM) - Consejo Nacional de Investigaciones Científicas y Técnicas (CONICET), Campus UNSAM, San Martín, Buenos Aires, Argentina; 7 Torrey Pines Institute for Molecular Studies, Port St. Lucie, Florida, United States of America; Federal University of São Paulo, Brazil

## Abstract

**Background:**

*Trypanosoma cruzi* ribosomal P proteins, P2β and P0, induce high levels of antibodies in patients with chronic Chagas' disease Cardiomyopathy (CCC). It is well known that these antibodies alter the beating rate of cardiomyocytes and provoke apoptosis by their interaction with β1-adrenergic and M2-muscarinic cardiac receptors. Based on these findings, we decided to study the cellular immune response to these proteins in CCC patients compared to non-infected individuals.

**Methodology/Principal findings:**

We evaluated proliferation, presence of surface activation markers and cytokine production in peripheral blood mononuclear cells (PBMC) stimulated with P2β, the C-terminal portion of P0 (CP0) proteins and *T. cruzi* lysate from CCC patients predominantly infected with TcVI lineage. PBMC from CCC patients cultured with P2β or CP0 proteins, failed to proliferate and express CD25 and HLA-DR on T cell populations. However, multiplex cytokine assays showed that these antigens triggered higher secretion of IL-10, TNF-α and GM-CSF by PBMC as well as both CD4+ and CD8+ T cells subsets of CCC subjects. Upon *T. cruzi* lysate stimulation, PBMC from CCC patients not only proliferated but also became activated within the context of Th1 response. Interestingly, *T. cruzi* lysate was also able to induce the secretion of GM-CSF by CD4+ or CD8+ T cells.

**Conclusions/Significance:**

Our results showed that although the lack of PBMC proliferation in CCC patients in response to ribosomal P proteins, the detection of IL-10, TNF-α and GM-CSF suggests that specific T cells could have both immunoregulatory and pro-inflammatory potential, which might modulate the immune response in Chagas' disease. Furthermore, it was possible to demonstrate for the first time that GM-CSF was produced by PBMC of CCC patients in response not only to recombinant ribosomal P proteins but also to parasite lysate, suggesting the value of this cytokine to evaluate T cells responses in *T. cruzi* infection.

## Author Summary

Chronic Chagas' disease Cardiomyopathy (CCC) is the most frequent and severe consequence of the chronic infection by protozoan parasite *T. cruzi*. Patients with CCC develop high levels of antibodies against ribosomal P proteins of *T. cruzi*, called P2β and P0. These antibodies can cross-react with, and stimulate, the β1-adrenergic and M2 muscarinic cardiac receptors, inducing a functional and pathological response in cardiomyocytes. In this study, we focused on the cellular immune response developed by CCC patients in response to *T. cruzi* ribosomal P proteins. Peripheral blood mononuclear cells (PBMC) from CCC patients stimulated with both proteins neither proliferated nor induced the expression of activation markers on CD4+ and CD8+ T cells. However, these cells responded by the secretion of IL-10, TNF-α and GM-CSF, giving evidence that there is indeed a pool of specific T cells in the periphery responsive to these proteins. Interestingly, the cytokines profile was not related with those described to whole parasite lysate or other recombinant proteins, suggesting that each parasite protein may contribute differently to the complex immune response developed in patients with Chagas' disease.

## Introduction

*Trypanosoma cruzi*, the etiological agent of Chagas' disease, affects approximately 8–10 million people, and its infection is one of the major human health problems in Central and South America, being extended now to Europe (especially Spain and Portugal), the United States, Canada, Japan and Australia [1], [2], [3]. Upon exposure to the parasite, the humoral and cellular immune responses elicited by the host, keep acute parasitemia under control [4], [5]. However, approximately 30–40% of the infected individuals, several years after initial exposure, develop clinical symptoms of visceral damage, which may include cardiac lesions, digestive alterations or both manifestations (cardiac plus digestive) [5]. Chronic Chagas' disease Cardiomyopathy (CCC), the most frequent and severe consequence of the chronic infection by *T. cruzi*, is manifested predominately as an arrhythmogenic cardiomyopathy [6]–[9].

Up to now, the mechanisms of the pathophysiology of Chagas' disease are not completely elucidated and two main hypotheses have been proposed. The first one is based on the inflammatory reaction elicited by the parasite leading to tissue damage, while the second argues for an autoreactive process resulting from an impaired immune response associated with molecular mimicry [10]–[13]. However, it is currently accepted that both mechanisms are not mutually exclusive and that Chagas' disease is the result of both, parasite persistence in the chronic phase and the presence of autoantibodies/self-reactive T cells to host molecules [14], [15]. As supporting evidence for the autoimmune hypothesis, previous work in our laboratory demonstrated the presence of circulating antibodies against ribosomal P proteins of *T. cruzi* (anti-P Abs) with agonist-like properties on cardiac receptors in patients with CCC [16]–[24]. Those Abs predominantly recognized the C-terminal end of P2β (peptide R13, EEEDDDMGFGLFD) or P0 proteins (peptide P015, EEEDDDDDFGMGALF), which bear structural similarity to the acidic motif, AESDE, located on the second extracellular loop of the cardiac receptor [19], [20], [22]. Several studies including patients with CCC as well as experiments performed in mice immunized with recombinant P2β or P0 protein demonstrated a correlation between the presence of anti-P Abs and cardiac disorders [21], [22]. These findings were confirmed by the generation of anti-R13 monoclonal Ab, mAb 17.2, which not only induce a dose-dependent increase on the beating frequency of rat cardiomyocytes in culture that is abolished by bisoprolol, a specific β1-adrenergic receptor antagonist [25], but also provoke apoptosis in the murine cardiac cell line HL-1 by its long-lasting β1-AR stimulatory activity [24]. The humoral immune response against ribosomal P proteins has been largely studied in patients with CCC; however, little is known about their recognition by T cells.

Most studies concerning the T cell immune response in Chagas' disease, have been performed using freshly isolated peripheral blood mononuclear cells (PBMC) but stimulated with epimastigote (the replicative form found in the midgut of insect vectors) or trypomastigote (the infective form found in the bloodstream and other human extracellular fluids) lysate [26]–[29]. Few investigations have been focused on the reactivity of T cells against purified antigens of the parasite [30]–[40]. To date, studies performed with recombinant parasite proteins, such as the cytoplasmatic repetitive antigen (CRA), B13, trans-sialidase, and paraflagellar rod proteins on PBMC and cruzipain on T cells lines revealed that patients with CCC produced significant amount of IFN-γ upon stimulation, which is in line with the typical pattern of inflammatory response described for *T. cruzi* lysate [34]–[40]. However, Lorena *et al.* also reported that the flagellar repetitive antigen (FRA) induced proliferation of PBMC by thymidine incorporation, but no difference was observed in IFN-γ and TNF-α secretion between patients with CCC and non-infected individuals [37]. The aim of this study was to analyze the cellular immune response developed in patients with CCC against *T. cruzi* ribosomal P proteins, knowing the existence of a cross-reactive component at the humoral level. The specificity of the response was analyzed by proliferation and cytokine production using multiplex technology because it allows to quantify a large spectrum of cytokines in the same cell culture supernatant. Results showed that *T. cruzi* ribosomal P proteins, specifically P2β and the C-terminal portion of P0 (CP0, 110 aa), did not induce the proliferation of PBMCs from CCC in a different manner than non-infected individuals. However, these antigens were able to induce the secretion of IL-10, TNF-α and GM-CSF by PBMC as well as both CD4+ and CD8+ T cells in patients with CCC. Surprisingly, ribosomal P proteins did not stimulate but actually reduced the secretion of IFN-γ in cardiac patients. Furthermore, our results demonstrate for the first time that GM-CSF is produced in response not only to parasite lysate but also to ribosomal P proteins. These findings suggest that GM-CSF production could be included in the future to evaluate whole parasite and parasite protein specific T cell responses in Chagas' disease.

## Methods

### Ethics statement

The research protocols followed the tenets of the Declaration of Helsinki and were approved by the Medical Ethics Committee of Ramos Mejía and Fernández Hospitals. All enrolled patients gave written informed consent, according to the guidelines of the Ethical Committee of the Hospitals, before blood collection and after the nature of the study was explained.

### Study population

Patient selection was conducted at the Cardiovascular Division of the Ramos Mejía and Fernández Hospitals, Buenos Aires, Argentina. Positive serology for Chagas' disease was determined by two or more tests (indirect immunofluorescence, enzyme-linked immunosorbent assay [ELISA], indirect hemagglutination, or complement fixation). Patients who had at least two of three tests were considered positive for Chagas' disease. Patients underwent a complete clinical and cardiologic examination that included medical history, physical examination, electrocardiogram (ECG) at rest, laboratory and chest X-ray analysis, and echo doppler cardiography evolution. The exclusion criteria included the presence of systemic arterial hypertension, diabetes mellitus, thyroid dysfunction, renal insufficiency, chronic obstructive pulmonary disease, hydroelectrolytic disorders, alcoholism, history suggesting coronary artery obstruction and rheumatic disease, and the impossibility of undergoing the examinations. The study population consisted of 27 patients who completed the screening protocol and were diagnosed with Chronic Chagas' disease Cardiomyopathy.

Twenty non-infected individuals (NI), within the same age range (30–70 years old) and showing negative serological tests for Chagas' disease, were included as control group.

### TSSA recombinant proteins

Due to its predominant clonal proliferation, the *T. cruzi* species is composed by multiple strains showing extensive genetic diversity, which were recently grouped into 6 evolutionary lineages or discrete typing units (DTUs) known as TcI to TcVI [41].

Gluthatione *S*-transferase (GST)-fusion proteins bearing the entire TSSA from Sylvio X-10/1 strain (henceforth TSSA Sy, representative of TSSA isoforms from DTU TcI parasites) and CL Brener strain (henceforth TSSA CL, representative of TSSA isoforms from DTUs TcII/TcV/TcVI parasites) were expressed in *Escherichia coli* BL21 strain and purified as described [42]. Briefly, supernatants of bacterial cultures transformed with the indicated construct were induced for 3 h at 28°C with 0.250 mM isopropyl—β-D-thiogalactopyranoside, purified by glutathione-Sepharose chromatography and extensively dialyzed against PBS. The purity and integrity of GST-TSSA samples was assessed with silver-stained SDS-PAGE gels [42].

### *T. cruzi* lineage identification by immunophenotyping

#### Enzyme-linked immunosorbent assay (ELISA)

Polystyrene microplates (Nunc Maxisorp, Roskilde, Denmark) were coated overnight at 4°C with 1 µg of either GST-TSSA Sy or GST-TSSA CL protein in 100 µL of carbonate buffer. Additional wells were coated with recombinant GST expressed and purified as stated above to detect sera background reactivity. Plates were washed with TBS and then blocked with TBS containing 4% non-fat dry milk (TBS-M) for 1 h at 37°C. After washing, 100 µL of each human serum (dilution 1/100 in TBS-M) was loaded onto plates and incubated for 1 h at 37°C. After washings with TBS, plates were incubated with 100 µl of HRP-conjugated goat anti-human Ig (dilution 1/10,000 in TBS-M) (Sigma, St Louis, MO, USA). Enzyme activity was revealed with TMB (Sigma, St Louis, MO, USA) and Optical Density (OD) was read at 450 nm with an Automated Plate Reader (Molecular Devices, CA, USA). All samples were tested in duplicate, in two independent experiments. Sera from 4 non-infected individuals were also included on the plate to determine the baseline level. Serum samples showing OD values below NI baseline value + 3 standard deviation (SD) were considered negative, while those rendering OD values between NI baseline value + 3 SD and NI baseline + 5 SD were considered non-conclusive.

#### Dot blot assays

A 1.5 µl drop with 1 µg of each GST-fusion protein was applied to a nitrocellulose filter (GE HealthCare, Uppsala, Sweden), allowed to dry at room temperature, blocked with TBS supplemented with 5% non-fat dry milk and incubated for 2 h with serum samples diluted 1/200. Washes were performed four times with TBS 0.2% Tween 20. Anti-human Ig Abs conjugated to HRP were diluted 1/5,000, incubated for 1 h in TBS 5% non-fat milk and revealed using either West-Pico or West-Fempto (both from Pierce, Rockford, USA) chemiluminescent substrates.

### Antigens

Whole antigenic lysate from *T. cruzi* epimastigotes was prepared as described previously [43]. Briefly, fresh epimastigotes (CL Brener, DTU Tc VI) cultured in a liquid medium (liver infusion tryptose), were collected by centrifugation and washed three times with PBS. After centrifugation at 500xg during 5 min, the parasites were resuspended in lysis buffer (PBS, EDTA 1 mM, β-mercaptoethanol 5 mM, 0.1% SDS and protease inhibitors cocktail) and submitted to three cycles of freezing-thawing. The parasite lysate was diluted with PBS at 1 mg/ml, filter sterilized on 0.2 µm-pore-size membranes, assayed for protein concentration, aliquoted, and stored at −80°C until use.

The *T. cruzi* recombinant proteins selected for this study were P2β-His and CP0-His; this last one corresponds to the C-terminal portion of P0 (110 aa). The ribosomal P proteins were obtained and purified by means as His_6_-tag as described [44]. The purity and specificity of the recombinant proteins were analyzed by SDS-PAGE gels and Western-blot with a pool of chagasic and non-infected sera. Protein concentration was determined by Bradford (BioRad, Hercules, CA, USA), using BSA (Sigma, St Louis, MO, USA) as standard protein.

Peptides were prepared by solid-phase method of Merrifield as described by Müller *et al*. with a semi-automatic multi-synthesizer NPS 4000 (Neosystem, Strasbourg, France) [45]. Their purity was assessed by High Performance Liquid Chromatography (HPLC) and identified by mass spectrometry. Peptide R13 (EEEDDDMGFGLFD) was derived from the 13 C-terminal amino acids of P2β, P015 (EEEDDDDDFGMGALF) from 15 C-terminal region of P0 protein, and peptide H13 (EESDDDMGFGLFD) was derived from the corresponding region of mammalian ribosomal P proteins [46]. For ELISA, these peptides were coupled at a molar ratio of 1∶30 to BSA (Sigma, St Louis, MO, USA) with 0.05% glutaraldehyde as previously described [45]. The products were assessed by analytical HPLC and amino acid analysis was used to calculate the peptide–BSA molar ratio.

### Enzyme-linked immunosorbent assay (ELISA)

Microwell plates (Nunc Maxisorp) were coated overnight at 4°C with 50 ng protein/well of *T. cruzi* lysate, 2 µg/well of recombinant proteins P2β-His and CP0-His or 2 µM of synthetic peptide in 50 µL of 0.05 M carbonate buffer pH = 9.6. Plates were washed with PBS containing 0.1% Tween-20 (PBST) and then blocked with PBST containing 2.5% non-fat dry milk (PMT) for 1 h at 37°C. After washing, 50 µL of each diluted human serum (dilution 1/200 in PMT) was loaded onto plates and incubated for 1 h at 37°C. Following washing, plates were incubated with 50 µl of peroxidase-conjugated goat anti-human IgG (dilution 1/3,000 in PMT) (Sigma, St Louis, MO, USA). Enzyme activity was revealed with TMB and, OD was read at 415 nm with an Automated Plate Reader (Molecular Devices, CA, USA). All samples were tested in duplicate. Sera from 8 non-infected individuals were also included on the plate to determine the baseline level, as the OD mean value +3 SD. Antibody level is expressed as Reactivity index which was determined as the OD mean value of each serum sample/baseline value.

### Cell preparation and proliferation assay

Peripheral blood mononuclear cells (PBMC) were isolated from heparinized blood by Ficoll-Hypaque density gradient centrifugation (GE HealthCare, Uppsala, Sweden), washed once and resuspended in RPMI-1640 medium containing 100 U/ml penicillin, 100 mg/ml streptomycin, 2 mM L-glutamine and 5% of AB Rh-positive heat-inactivated normal human serum (Sigma, St Louis, MO, USA). Cell suspensions (200 µl) were cultured as triplicates in the presence or absence of different stimuli for 4 or 6 days at a density of 2.5×10^5^ cells/well in 96-well sterile plates (round bottom). Stimuli used in the cultures included *T. cruzi* lysate, P2β-His, CP0-His (at a final concentration of 10 µg/ml for 6 days), peptides R13, P015 and H13 (at a final concentration of 5 µg/ml for 6 days) while PHA (Phitohemaglutinin, Sigma, at a final concentration of 5 µg/ml for 4 days) was used as positive control. All concentrations were determined by performing titration experiments. After the incubation period, cultures were exposed to 1 μCi/well of ^3^H-thymidine (^3^H-TdR, specific activity, 2 Ci/mmol, Amersham, Arlington Heights, IL) for 6 h and then harvested on glass fiber filters. The incorporated radioactivity was determined by liquid scintillation counting. All cultures were performed in triplicate. Results are expressed as Stimulation Index, calculated as the mean cpm of stimulated cultures/mean cpm of non-stimulated (culture medium only) cultures.

### Phenotypic analysis of PBMC

2.5×10^6^ cells were cultured in 24-well plates in 1 ml cultures for 6 days with either medium alone, or *T. cruzi* lysate, P2β-His, CP0-His (at a final concentration of 10 µg/ml). After centrifugation, cells were washed, resuspended in ice-cold PBS, stained for 30 min at 4°C with the following fluorescent-labeled monoclonal antibodies: allophycocyanin (APC) conjugated anti-CD3 + phycoerythrin-cyano dye Cy5 (PE-Cy5) conjugated anti-CD4 + phycoerythrin (PE) conjugated anti-HLA-DR + fluoresceinisothiocyanate (FITC) conjugated anti-CD25, or APC anti-CD3 + PE-Cy5 anti-CD8 + PE anti-HLA-DR + FITC anti-CD25. Cells were then fixed with 4% formaldehyde in PBS and kept at 4°C until analyzed by flow cytometry. In all cases, 10,000 to 15,000 events in the lymphocyte gate were acquired using a FACSAria flow cytometer (Becton Dickinson). Phenotypic analyses were carried out with FlowJo flow cytometric analysis software (TreeStar), selecting the small lymphocyte population. PBMC stained with FITC, PE-, APC- and PE-Cy5- labeled Ig control Abs were included in all experiments for background fluorescence. All Abs were purchased from BD Biosciences (San Diego, CA, USA).

### Isolation of CD3+CD4+ and CD3+CD8+ T cells from whole PBMC samples

CD8+ T cells were isolated from PBMC by positive selection using EasySep CD8 Selection Kit (StemCell Technologies, Inc., Vancouver, Canada), while CD4+ T cells were separated from CD3+CD8^neg^ T cells by negative selection (EasySep CD3 Selection Kit, StemCell Technologies). The purity of both populations was assessed by flow cytometry using specific conjugated mAb (see “Phenotypic analysis of PBMC”) and, it was shown to be higher than 90% for both T cells subsets.

### Cytokine determination by multiplex technology

IL-2, IL-4, IL-10, IL-13, IL-17, IFN-γ, GM-CSF and TNF-α were measured in the supernatants of whole PBMC cultures stimulated in the presence or absence of the indicated antigens and collected on days 1, 2 and 6 after stimulation. In addition, the same cytokines were quantified in cultures of isolated CD4+ or CD8+ T cells (5×10^5^ cells) co-cultured with irradiated CD3^neg^ T cells (ratio 1∶1) in the presence or absence of antigen after 6 days of stimulation. Cytokines were measured by using MILLIPLEX MAP Human Cytokine/Chemokine Kit (for 8 cytokines) following the manufacturer's directions (Millipore, St Charles, MO) and Luminex instrument and Beadlyte software were used for analysis. All samples were tested in duplicate. Results are expressed in ng/ml or Fold increase (FI) which was determined as [(cytokine in stimulated culture) - (cytokine in NS culture)]/(cytokine in NS culture), where NS denotes non-stimulated cultured PBMC.

### Data analysis

Statistical analysis was performed with GraphPad Prism statistical software (GraphPad Software). The nonparametric Mann-Whitney U test was used to generate *P* values comparing the median experimental values between groups each of the multiple sets of experimental data. Within each experiment, overall statistical significance of each result at both 10% and 5% significance was determined using Holm-Bonferroni Correction. Differences were considered statistically significant at *P*<0.05.

## Results

### Characteristics of patients with chronic Chagas' disease Cardiomyopathy

Patients included in this study were all born in endemic areas from Argentina and Bolivia, and at the time of the enrollment they have been living in Buenos Aires (where no vectorial transmission occurs) for more than ten years, in average. The mean age was 54.2±10.1, and 57% were female.

All *T. cruzi*-infected subjects were in the chronic phase of Chagas' disease, involving only cardiac alterations. According to the New York Heart Association (NYHA) functional classification system, patients were classified as Class I, II, III/IV. Patients with no functional limitations but with some electrocardiographic alterations were classified as Class 0 [5].

Blood samples yielded negative results for currently used PCR protocols targeting parasite DNA [47], which is frequently the case in chronic chagasic patients due to low parasitemia. Taking this into account, we analyzed the profile of the humoral anti-TSSA (trypomastigote small surface antigen) response in our study patients as an indirect means of identifying the genotype of the infecting strain(s) [48], [49]. To carry out this analysis, we evaluated the reactivity of serum samples against either TSSA Sy (the TSSA isoform from DTU TcI) or TSSA CL (the TSSA isoform from DTUs TcII/V/VI) in conventional ELISA and dot-blot (see Text S1 for details and Figure S1).

The main characteristics of the study population are summarized in Table 1.

**Table 1:** Characteristics of patients with chronic Chagas' disease Cardiomyopathy.

Patient Code	Gender^a^	Age	Nationality	Clinical classification (NYHA)^b^	*T. cruzi* Ab level (1/dilution at OD = 1.0)^c^	*T.cruzi* lineage^d^
P8	F	63	Bolivia	II	200	VI
P10	M	70	Argentina	0	300	ND
P11	M	34	Argentina	0	2,000	VI
P14	F	53	Argentina	I	2,000	VI
P19	F	52	Argentina	I	<200	ND
P20	F	63	Argentina	II	1,600	ND
P21	M	56	Argentina	III/IV	4,000	VI
P22	F	53	Bolivia	I	2,000	VI
P23	M	42	Argentina	I	1,100	I
P24	F	56	Argentina	I	4,500	VI
P25	M	47	Argentina	II	850	VI
P26	F	62	Bolivia	II	26,000	VI
RM1	F	47	Argentina	I	4,000	VI
RM2	M	65	Argentina	0	100	VI
RM4	F	52	Bolivia	0	9,500	I
RM5	F	37	Bolivia	II	10,000	VI
RM6	F	56	Argentina	II	8,000	I
RM7	F	54	Argentina	0	5,000	VI
RM8	F	66	Argentina	0	300	VI
RM9	F	46	Bolivia	II	15,000	VI
RM10	M	50	Bolivia	0	7,000	VI
RM11	M	59	Bolivia	0	8,000	VI
RM12	F	67	Bolivia	II	500	I-VI
RM13	M	40	Argentina	0	3,000	VI
RM14	F	60	Bolivia	II	12,000	VI
RM15	M	64	Argentina	0	2,000	VI
RM16	M	42	Bolivia	0	6,000	VI

a F: female, M: male.

b Patient's heart failure was classified according to New York Heart Association (NYHA) Functional Classification. Class I: Patients with cardiac disease with slight functional alterations but resulting in no limitation of ordinary physical activity; however, elevated activity causes symptoms, such as fatigue, palpitation, or dyspnea (shortness of breath); Class II: Patients with cardiac disease resulting in slight limitation of physical activity; comfortable at rest, but ordinary physical activity results in symptoms; Class III: Patients with cardiac disease resulting in marked limitation of physical activity; comfortable at rest, but less than ordinary activity causes symptoms; Class IV: Patients with cardiac disease resulting in marked limitation of physical activity, unable to carry out any physical activity without discomfort; symptoms of cardiac insufficiency at rest.

Patients with cardiac disease without any functional alteration were classified as Class 0.

c The level of antibodies directed to *T. cruzi* lysate was determined by in-house ELISA as described upon Methods.

d *T cruzi* lineage was analyzed by TSSA recognition as described upon Methods. Results are shown in Figure S1. ND: not detected.

### Humoral immune response against ribosomal P proteins

To characterize the humoral response in the subject population included in this study, the antibody reactivity against *T. cruzi* lysate, ribosomal P proteins, P2β and CP0, together with their C-terminal peptides R13 and P015 was determined in sera of CCC patients and non-infected individuals by ELISA. The reactivity against peptide H13, which corresponds to the C-terminal region (residues 102–115) of the human ribosomal P protein was also measured.

Results showed that sera from CCC patients presented reactivity against *T. cruzi* lysate, with titers ranging from 1/200 to 1/20,000 (Table 1). Only patient P19 showed a titer against parasite proteins similar to those detected in non-infected individuals (<1/200 at OD = 1). Although antibodies in the serum sample from this patient were not detected by in-house ELISA, two of three serological tests for *T. cruzi* infection, together with clinical and cardiological examinations confirmed patient P19 to have CCC. In addition, sera of all patients, including P19, reacted with a broad range of *T. cruzi* proteins as determined by Western-blot (data not shown).

**Figure 1:**
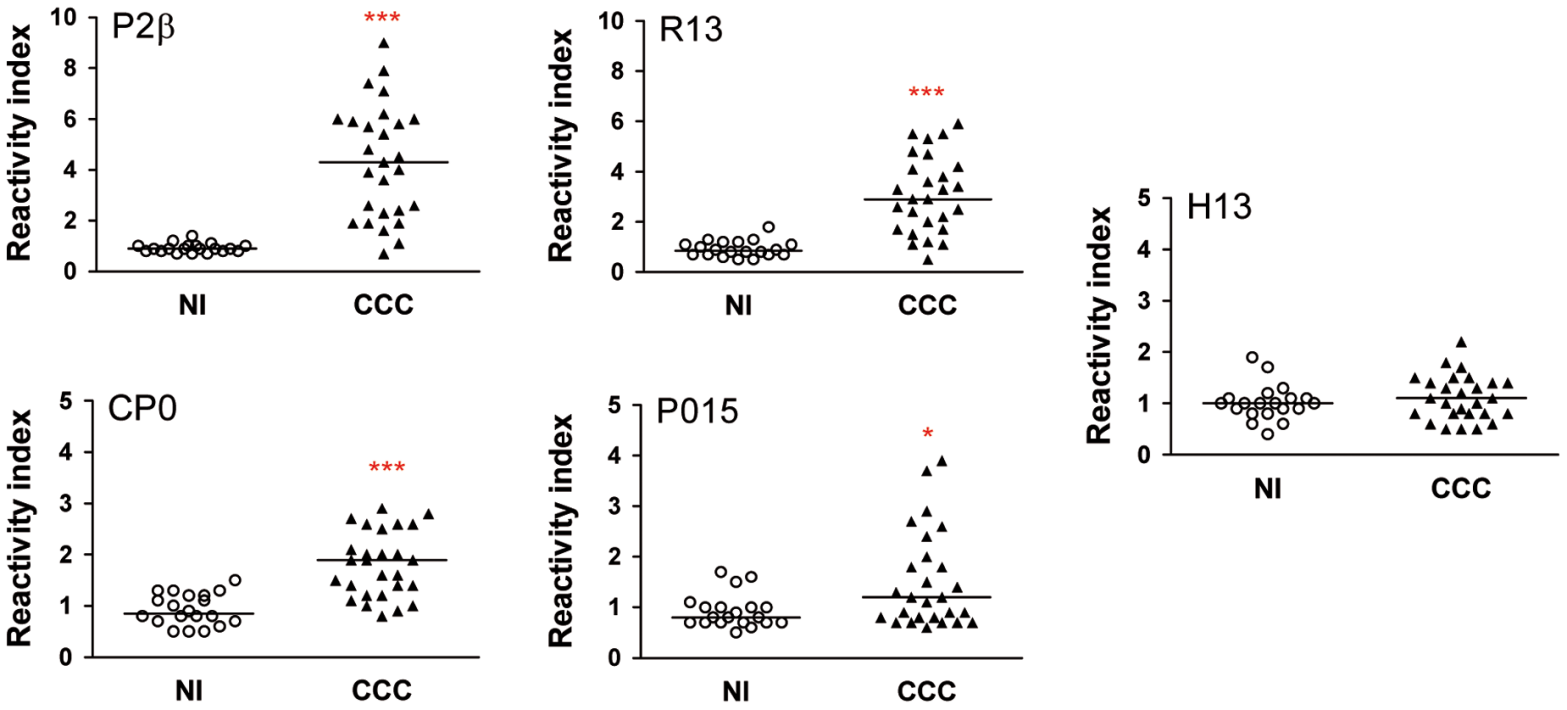
Humoral response against ribosomal P proteins and their C-terminal peptides. The presence of antibodies directed against P2β and CP0 proteins as well as peptides R13, P015 and H13 in the sera of 27 patients with chronic Chagas' disease Cardiomyopathy patients (CCC) and 20 non-infected individuals (NI) was determined by ELISA as described under Methods. Results are expressed as Reactivity index, calculated as: (Optical Density mean value obtained of each serum sample/baseline value). Each symbol represents data from a single subject. Statistical analysis was performed using the Mann-Whitney U Test, ***P<0.001, *P<0.05. The line for each of the scatters represents the median.

The majority of CCC patients (24/27) showed reactivity (Reactivity index >1.7) to ribosomal P2β protein and its peptide R13. The level of anti-CP0 antibodies was also elevated in the chagasic patients (17/27) compared to non-infected individuals, but the overall reactivity was lower than that observed for P2β protein (Figure 1). On the other hand, only marginal differences were determined in the median of the Reactivity Index for the anti-P015-antibodies in cardiac patients in comparison with non-infected subjects. No difference was observed against peptide H13 (human P ribosomal protein derived) between both groups of individuals (Figure 1). Together, these results showed that CCC patients mount a significant antibody response to ribosomal proteins as well as to peptides R13 and to a lower level to P015 in comparison to non-infected subjects.

### Patients with chronic Chagas' disease Cardiomyopathy and non-infected individuals showed similar proliferative profiles in response to ribosomal P proteins

In order to investigate the cellular response to ribosomal P proteins, PBMC from CCC patients and non-infected individuals were tested for their proliferative capacity in response to different *T. cruzi* antigens. To determine the optimal protein and peptide concentration yielding the most consistent results, the proliferative response was initially assayed in PBMC cultures from 4 cardiac patients non-included in this study. The results showed that 10 µg/ml of *T. cruzi* lysate or ribosomal P proteins and 5 µg/ml of the peptides were optimal to trigger proliferative responses, and so these concentrations were used in the studies presented here.

**Figure 2:**
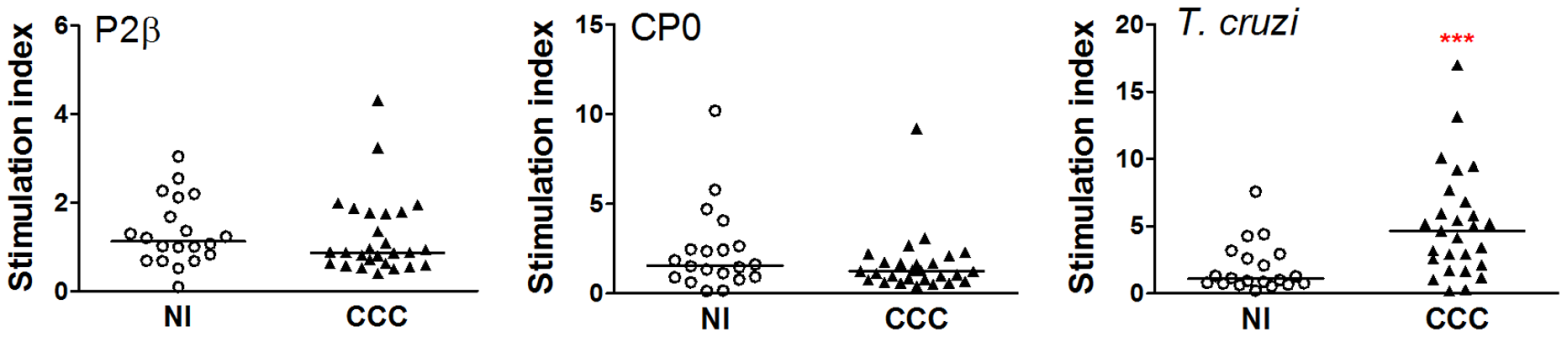
Parasite lysate but not ribosomal proteins trigger PBMC proliferative responses. PBMC isolated from chronic Chagas' disease Cardiomyopathy patients (CCC; n = 27) and non-infected individuals (NI; n = 20) were seeded at 2.5×10^5^ cells/well and stimulated with *T. cruzi* lysate, P2β or CP0 proteins (10 µg/ml) or medium alone for 6 days. Cell proliferation was determined by ^3^H-thymidine incorporation. Results are expressed as Stimulation index, calculated as: (mean cpm of stimulated cultures/mean cpm of non-stimulated cultures (medium only)). Each symbol represents data from a single subject. Statistical analysis was performed using the Mann-Whitney U Test, ***P<0.001.

As shown in Figure 2, the majority of PBMC from CCC patients proliferated upon stimulation with *T. cruzi* lysate (Stimulation index median: 4.45) compared to PBMC from non-infected individuals (Stimulation index median: 1.07; *P<*0.001). On the contrary, the stimulation index of PBMC from cardiac patients and control subjects in response to ribosomal P proteins (Figure 2) as well as to peptides R13, P015 and H13 was not significantly different (data not shown). PBMC from all subjects proliferated in response to PHA and the responses were not significantly different between the cardiac and non-infected individuals (data not shown).

**Figure 3:**
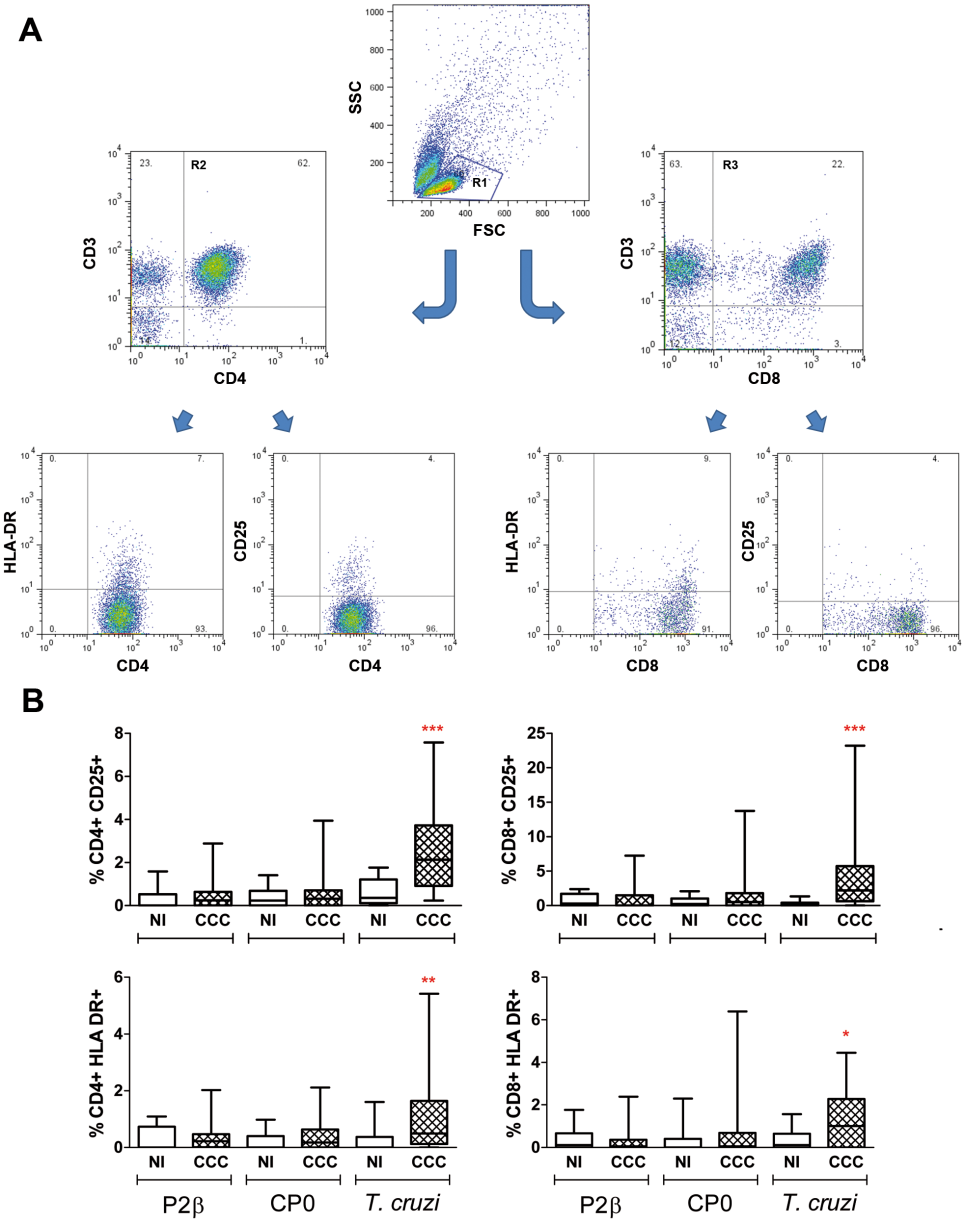
Activation markers on CD4+ and CD8+ T cell subsets upon *T. cruzi* and ribosomal protein activation. PBMC isolated from patients with chronic Chagas' disease Cardiomyopathy (CCC; n = 27) and non-infected individuals (NI; n = 20) were seeded at 2.5×10^6^ cells/well and stimulated with *T. cruzi* lysate, P2β or CP0 proteins (10 µg/ml) or medium alone for 6 days. PBMC were stained with CD3-APC, CD4-PE-Cy5 or CD8-PE-Cy5 and activation marker-specific labeled antibodies (CD25-FITC and HLA-DR-PE) prior to flow cytometry analysis. 10,000–15,000 events in the lymphocyte gate (R1 gate) were acquired using a FACSAria flow cytometer (Becton Dickinson); dead cells were excluded by forward *vs* side-scatter (FSC/SSC) gating. A) Gate-pathway used to determine the activation expression in the populations graphed in B. B) Results were expressed as the percentage of CD25+ or HLA-DR+ cells in CD3+CD4+ (R2 gate) or CD3+CD8+ (R3 gate) lymphocytes. Horizontal lines represent the median and percentiles 25–75th, vertical lines represent percentiles 5–95th. Statistical analysis was performed using the Mann-Whitney U Test, ***P<0.001, **P<0.01, *P<0.05.

To characterize the phenotype of the cells after the stimulation with the different stimuli, cells were stained with different T cell markers and analyzed by flow cytometry. The forward *vs* side scatter dot plots revealed that the frequency of lymphocyte population in non-stimulated cultures was significantly lower in cardiac patients compared with non-infected individuals (48±13% *vs* 62±10%, respectively; *P*<0.001). However, the CD3+CD4+:CD3+CD8+ ratio was approximately 2∶1 in both groups. Interestingly, results showed that CCC patients present higher subsets of CD25 and HLA-DR positive cells on both CD3+CD4+ and CD3+CD8+ populations upon *T. cruzi* stimulation (Figure 3). However, the expression of these markers was similar in T cells from cardiac patients and non-infected individuals when cells were stimulated with ribosomal P proteins (Figure 3).

### Cytokine response to ribosomal P proteins

**Figure 4:**
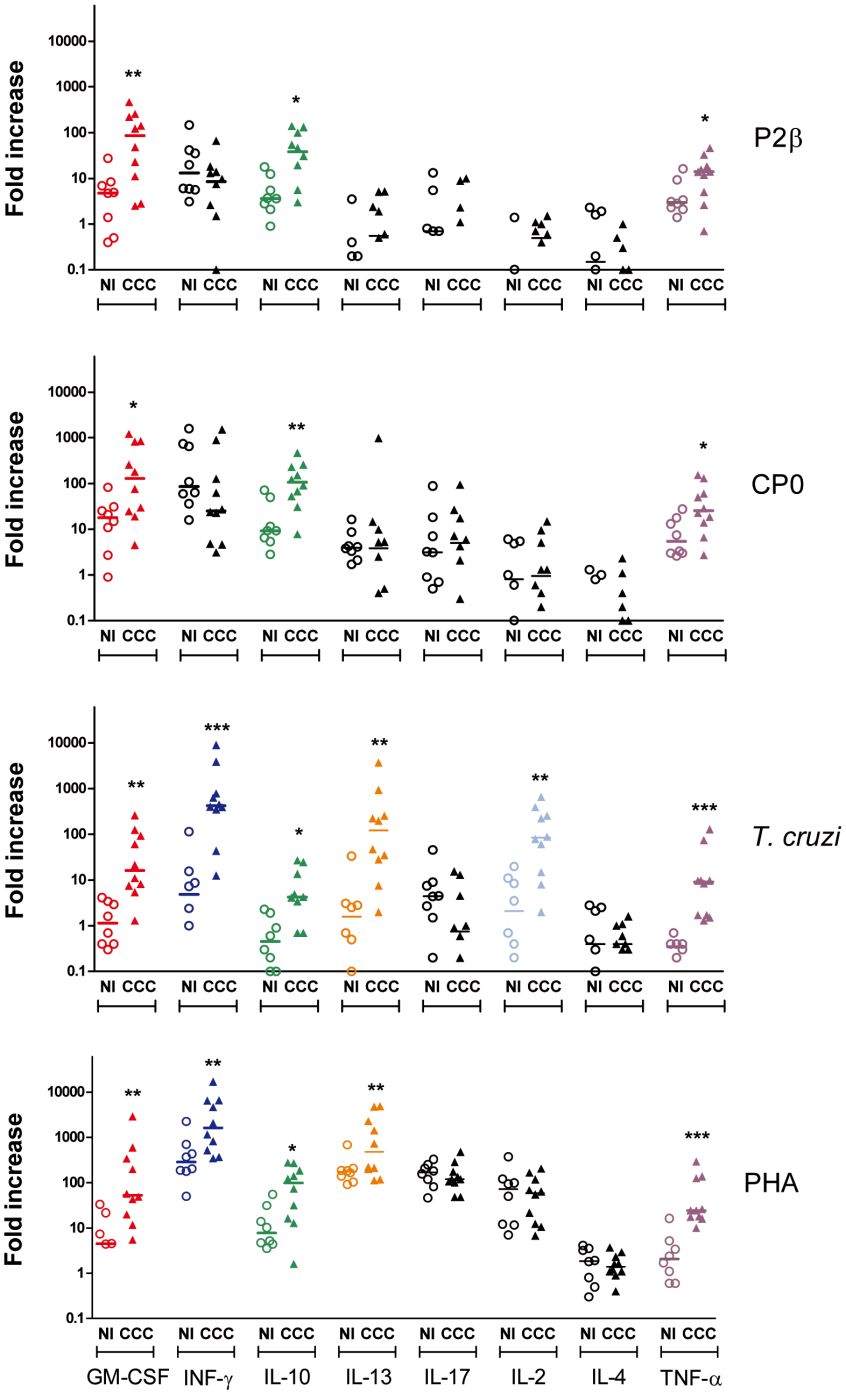
Cytokine expression in PBMC from chagasic patients after in vitro stimulation. PBMC isolated from patients with chronic Chagas' disease Cardiomyopathy (CCC; n = 10) and non-infected individuals (NI; n = 8) were cultured in the presence of the indicated stimulus. Supernatants were collected on days 1, 2 and 6 and cytokines were quantified by multiplex technology. The Fold increase (FI) was calculated as: [(cytokine in stimulated culture) - (cytokine in NS culture)]/(cytokine in NS culture), where NS denotes non-stimulated cultured PBMC. The maximum FI out of the 3 day-determinations for each subject and for each cytokine are shown. In color are denoted the cytokines for which the FI in the CCC patients were statistically significantly higher than in non-infected individuals. Each symbol represents data from a single subject. Statistical analysis was performed using the Mann-Whitney U Test, ***P<0.001, **P<0.01, *P<0.05.

Given the lack of proliferative response to ribosomal P proteins in the CCC patients, T cell activation was studied by analyzing cytokine secretion. Thus, PBMCs from 10 cardiac patients with different disease severity, and 8 non-infected donors were stimulated with P2β and CP0 proteins and *T. cruzi* lysate as well as PHA as positive control. Supernatants after 1, 2 and 6 days post-stimulation were collected and multiplex analysis was performed to evaluate the levels of GM-CSF, IFN-γ, IL-10, IL-13, IL-17, IL-2, IL-4 and TNF-α. Despite the fact that cytokine responses have been studied by others after *T. cruzi* stimulation in patients with Chagas' disease [50], [51], reports have used different assays and stimulation/culture conditions making the direct comparison of all the cytokines difficult to achieve. In this study, we aimed to simultaneously evaluate the kinetic responses of multiple cytokines in the same culture well. Figure 4 shows the maximum fold increase detected for each cytokine and in each subject among day 1, 2 and 6 determinations. The fold increase was determined by the difference between cytokine production (in pg/ml) in stimulated wells and the cytokine production in non-stimulated control wells divided the cytokine production in non-stimulated control wells. The actual fold increase for each of the days and the background production in pg/ml of each of the cytokines in non-stimulated wells are shown in Figures S2 to S5 and S6, respectively.

Upon stimulation with ribosomal P proteins, GM-CSF, IL-10 and TNF-α were secreted at higher levels in cardiac patients compared with non-infected individuals (Figure 4 and Figure S2 and S3). However, both proteins induced similar levels of IFN-γ production in PBMC from cardiac patients and non-infected subjects (Figure 4). Furthermore, the fold increase of IFN-γ production in response to both proteins was lower and statistically significant in the cardiac group after only the first days post-stimulation (Figure S2 and S3). The level of IL-2, IL-4, IL-13 and IL-17 secreted after stimulation with the ribosomal P proteins was very low or null at any of the 3 time points analyzed and, it was found to be similar between CCC patients and non-infected individuals (Figure 4 and Figure S2 and S3).

A larger number of cytokines were produced in response to *T. cruzi* lysate or the universal stimulus PHA than in response to the individual ribosomal P proteins (Figure 4). Indeed, PBMC from cardiac patients in response to *T. cruzi* lysate also secreted statistically significant and higher levels of IFN-γ, IL-2 and IL-13 compared with non-infected individuals. IFN-γ and IL-13 were also increased in CCC patients *vs* non-infected individuals when PHA was used for stimulation. These results indicate that although the cells were capable of producing IFN-γ and IL-13 in response to whole parasite or PHA, their production was not detected when the ribosomal P proteins were used as stimulus. The kinetic cytokine profile for *T. cruzi* lysate and PHA is shown in Figure S4 and S5.

### Profiles of cytokine production by CD4+ and CD8+ T cell subsets derived from CCC patients

The results presented above revealed a cytokine signature expression upon stimulation with ribosomal P proteins and *T. cruzi* lysate in whole PBMC. To better understand the specific contribution of the T cells to this profile, CD3+CD4+ and CD3+CD8+ T cell subsets from three cardiac patients were enriched from PBMC and stimulated with the antigens in the presence of autologous antigen-presenting cells. Samples from patients RM11, RM12 and RM14 were chosen since they were among those that showed clear cytokines response after ribosomal P proteins stimulation.

**Figure 5:**
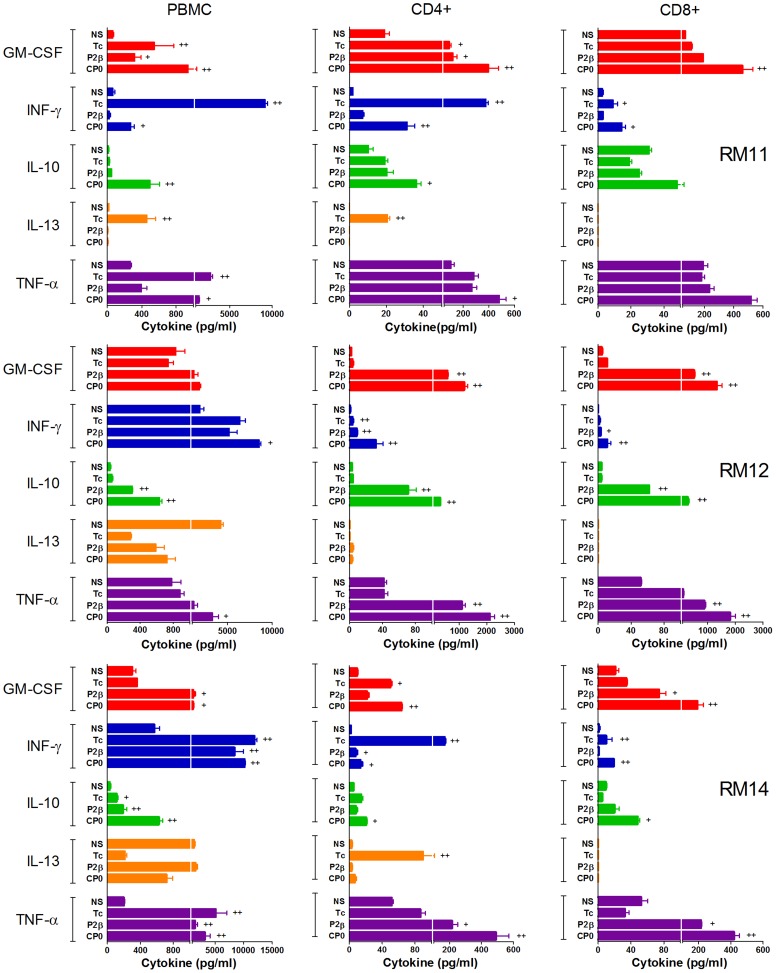
Cytokine production profile by CD4+ and CD8+ T cells derived from chagasic patients. CD4+ and CD8+ T cells from three chronic Chagas' disease Cardiomyopathy patients, called RM11, RM12 and RM14, were isolated as described under Methods. Enriched CD4+ or CD8+ T cells were co-cultured with the autologous CD3^(neg)^ fraction as antigen presenting cells and the indicated stimulus. Supernatants were collected on day 6 and cytokines quantified by multiplex technology. For comparison, results from whole PBMC from each of the patients are represented in the left panel. Symbol (+) indicates a fold induction between 3 to 5 and (++) a fold induction>5. The fold induction was calculated as: [(cytokine in stimulated culture) - (cytokine in NS culture)]/(cytokine in NS culture), where NS denotes non-stimulated cells.

As shown in Figure 5, GM-CSF was overall produced by both, CD4+ and CD8+ subsets by the 3 patients in response to the proteins and *T. cruzi* lysate. In general, IFN-γ was produced at very low levels by CD4+ and CD8+ T cells in all patients in response to the proteins, but enough to be different from the non-stimulated wells in the case of CD4+ T cells (Figure 5). IL-10 was found to be secreted most frequently by both T cell subsets. IL-13 was not produced by CD8+ T cells in any of the 3 patients analyzed and in response to all the stimuli tested. However, IL-13 was produced by CD4+ T cells in response to *T. cruzi* lysate and/or the proteins in the 2 of the 3 patients (RM11 and RM14). TNF-α was produced by both, CD4+ and CD8+ T cells and its production was higher in response to the proteins than to *T. cruzi* in 2 of the 3 patients. IL-2, IL-4 and IL-17 were not detected in response to any of the stimuli (data not shown).

## Discussion

Since it has been widely demonstrated the relevance of antibodies directed to ribosomal P proteins in the pathophysiology of Chagas' disease [21], [23], [24], this study aimed to further understand the cellular immune response raised against these proteins in CCC patients. Our results showed that PBMC did not proliferate upon *in vitro* stimulation with P2β and CP0 proteins. Additionally, the lack of proliferation in response to the proteins was associated with the absence of the expression of activation markers CD25 and HLA-DR on CD4+ and CD8+ T cell populations. These findings were also protein-specific, since *T. cruzi* lysate provoked an augmentation of both markers on the surface of T cells in agreement with data published by others [50], [51]. Interestingly, the percentage of both T cell subtypes, CD3+CD4+ and CD3+CD8+ in PBMC were similar in cardiac patients and non-infected individuals independently of the stimulus. These results suggest that the lack of proliferative response was not due to an overall decrease on the size of the T cell population, nor to a shutdown of the proliferative capacity in these patients since the same cells responded to *T. cruzi* lysate and a T cell specific universal mitogen such as PHA. However, it was possible to speculate that T cells specific to these proteins have been deleted by negative selection due to the similarity to the host specificities. In this regards, the analysis of the T cell response by cytokine release discarded this possibility since indeed, several cytokines were expressed in response to ribosomal *T. cruzi* proteins.

The use of multiplex technology allowed us to simultaneously analyze 8 cytokines, namely, IL-2, IL-4, IL-10, IL-13, IL-17, IFN-γ, TNF-α and GM-CSF, corresponding to well-described CD4+ and CD8+ associated cytokines. In particular, GM-CSF was included because not only its production has been associated to antigen mediated activation of T cells by us and others but also, the threshold of antigen requirement for its production is lower than for other cytokines as TNF-α, IL-4 or IFN-γ [52]–[54].

Our results showed that PBMC from CCC patients secreted high levels of GM-CSF, IL-10 and TNF-α in response to P2β and CP0 proteins. Interestingly, the secretion of IFN-γ at day 1 and 2 post-stimulation with ribosomal P proteins was similar or lower in cardiac patients *vs* non-infected individuals. Moreover, our data demonstrated that patients with CCC developed a different cytokine profile in response to *T. cruzi* and PHA stimulation than non-infected subjects.

Even though the secretion of GM-CSF, IL-10 and TNF-α in response to the proteins was significantly higher in cardiac *vs* non-infected individuals (*P*<0.05, nonparametric Mann-Whitney U Test), these *P* values nonetheless did not stay significant at the 5% level when a multiple comparison (all 32 cytokine/stimulus pairings) was performed by using Holm-Bonferroni correction. In contrast, the *P* values for these cytokines in response to *T. cruzi* lysate did reach statistical significance at the 5% level. This difference could be explained by the fact that the frequency of single specific parasite protein T cells within the bulk population is lower than the frequency developed in response to whole *T. cruzi* lysate and therefore it leads to lower cytokine secretion levels. However, it is important to remark that were the *P* values distributed at random amongst the proteins data, there would be only a 
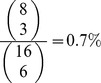
 chance of the three exact same cytokines (GM-CSF, IL-10 and TNF-α) being secreted in response to both proteins, demonstrating that the difference observed between cardiac patients and non-infected individuals was not a mere coincidence.

Following with *T. cruzi* lysate response, we observed that all studied cytokines were elevated and significantly different in the supernatants of cultured PBMC from cardiac patients with exception of IL-4 and IL-17. Upon PHA stimulation, PBMC from cardiac patients secreted higher amount of GM-CSF, IFN-γ, IL-10, IL-13, and TNF-α; similar production was observed for IL-2, IL-4 and IL-17 between both groups of individuals. In addition, and independently of the stimulus, our results also showed that these cytokines were secreted by both T cells populations, except for IL-13 which was predominantly produced by CD4+ T cells. Despite this finding, it is well-known that non-T cells, such as monocytes or B cells, also participate in the secretion of these cytokines. Indeed, Gomes *et al*. [28], by intracellular cytokine staining, reported that the majority of the IL-10-producing cells are monocytes (CD14+ cells) in asymptomatic patients, and the same group recently demonstrated that CD19+ B cells is another important source of this cytokine in cardiac patients [55].

Furthermore, the spontaneous release of cytokines in non-stimulated PBMC, which provides information about the basal level of cytokine production *in vivo*, showed a lower level for IFN-γ, IL-10, IL-13, and TNF-α in CCC patients (Figure S6). It should be mentioned that Dutra *et al.* demonstrated that the expression of IFN-γ, IL-10, IL-13 mRNAs was increased in PBMC from chagasic patients [33]. However, this discrepancy could depend either on the use of *ex vivo* PBMC or on the methodology used to determine cytokine expression. Our data, together with those reported by Giraldo *et al.* [56], may suggest that *T. cruzi* persistence provokes a general dysfunction in peripheral T cell response.

The high levels of pro-inflammatory cytokines, like IFN-γ and TNF-α, together with undetectable IL-4 production in response to PHA and *T. cruzi* stimulation suggest that there is a shift towards polarized Th1-type of cytokine response in CCC patients. Although IL-10 was first described related to Th2 cells, now is known that is produced by all T cells, including Th1 and a regulatory T cell subsets, called Tr1 cells or IL-10-producing cells [57]. Recent studies with an experimental murine model revealed not only the protective role of IL-10 against fatal myocarditis, but also demonstrated that this cytokine was produced by both CD4+ and CD8+ subsets of IFN-γ+IL-10+ double-producing T cells [58]. Similar data were obtained in studies by Belkaid *et al.* [59], where the main source of IL-10 in dermis and draining nodes of mice infected with *Leishmania major* is a subset of CD4+ T cells that produce both IL-10 and IFN-γ.

Studies performed with others recombinant parasite proteins demonstrated that the majority of chagasic patients develop a strong humoral and cellular immune response with a tendency to the typical pattern of inflammatory response described for *T. cruzi* lysate [34]–[40]. On the contrary, the cytokines released upon ribosomal P proteins stimulation made difficult to set a specific Th cells responsible for their secretion. This mixed cytokine profile which could be involved in balancing heart tissue damage and parasite persistence during chronic disease, strengthens in part the fact that B cells, through antibodies directed against P2β and CP0 and not T cells, would have the major role in the development of cardiac symptoms by their interaction with β1-adrenergic and M2 muscarinic receptors.

Interestingly, GM-CSF was secreted at high levels by PBMC from CCC patients when *T. cruzi* lysate, and both ribosomal P proteins were used as stimulus. To our knowledge, this is the first time that GM-CSF is used to evaluate the *T. cruzi* specific response of stimulated PBMC from cardiac patients. Instead, GM-CSF has been associated to a decrease in the rate of infection of both non-activated and IFN-γ activated macrophages infected with *T. cruzi* [60]. Moreover, Olivares Fontt *et al.* reported that the administration of exogenous recombinant GM-CSF improved the deficient immune response of chronically infected mice or, if neutralized by Ab anti-GM-CSF, it aggravated infection increasing parasitemia and host mortality in *T. cruzi* infected BALB/c mice [61]. In the aforementioned report, the role of GM-CSF was studied by correlating the outcome of infection with the titer of GM-CSF in plasma levels [61]. Even though it was not defined which cells were involved in GM-CSF secretion, it was speculated that lymphocytes could be in part contributing to the low but sustained amount of GM-CSF levels in infected mice. In our experiments, CD4+ and CD8+ T cells contributed almost equally with the secretion of this cytokine, independently of the stimulus, but it is not possible to discard that other cells as part of the PBMC pool also produced this cytokine.

While many questions remain regarding the pathogenesis of Chagas' disease, this study represents one of the most comprehensive about the cytokine profile in response to *T. cruzi* and two recombinant proteins, like P2β and CP0. The results show that a pool of PBMC in CCC patients has specificity for *T cruzi* proteins and that this specificity is revealed by a Th1-cytokine dominant milieu, combined with regulatory cytokines like IL-10 and IL-13. This observation reinforces the idea that a delicate cytokine equilibrium prevails during the chronic phase of the disease. Interestingly, another cytokines, namely GM-CSF, were found significantly increased in cardiac compared to non-infected individuals, tempting us to suggest that this cytokine may be further applied for studying antigen responses at different stages of the disease.

Finally, due to the limited number of patients infected with TcI (3/27) compared with TcVI (20/27), it was not possible to determine a correlation between the intensity of humoral and cellular immune response and the *T. cruzi* lineage detected by TSSA reactivity. Further studies in that sense would provide valuable information on the role and contribution of genetic variability of *T. cruzi* to the immune response developed in humans.

As a whole, our findings also demonstrate that not all parasite proteins provoke a strong T cell activation combined with a pattern of cytokines similar to those described to *T. cruzi* lysate or by infection with trypomastigote in CCC patients. In addition, as it was recently reported in the context of B cell-T cell recognition for other molecule-specificities [62], [63], it is possible to hypothesize that B cells specific for ribosomal P proteins could obtain help from T cells exhibiting different antigen reactivity. However, the interacting elements for T cell help recognition and activation may be the same for P2β and CP0, since a positive correlation was observed between the cytokines secreted by each of them (Figure S7). Currently, we are in the process of analyzing the immunoprevalence of recognition of these ribosomal P proteins and novel specificities involved in the immune response to *T. cruzi* infection in a large number of infected subjects at different stages of the disease.

## Supporting Information

Figure S1 ***T. cruzi*** **immunophenotyping of chagasic patients using TSSA proteins.** The presence of antibodies directed against GST, GST-TSSA Sy or GST-TSSA CL proteins was determined by ELISA in chronic Chagas' disease Cardiomyopathy patients (CCC) and non-infected individuals (NI) as described under Methods. Results are expressed as means of duplicates. Negative samples were those rendering OD values below NI baseline value ±3 SD (dark grey zone). Non-conclusive samples were those rendering OD values between NI baseline value ±3 SD and NI baseline ±5 SD (light grey zone). Samples indicated to the right were re-assayed by dot-blot revealed using West-Fempto except for patient P26, which was revealed using West-Pico chemiluminescent substrate.
(TIF)

Figure S2 **Cytokine kinetics in PBMC stimulated with P2β protein.** PBMC from patients with chronic Chagas' disease Cardiomyopathy patients (CCC; n = 10) and non-infected individuals (NI; n = 8) were cultured in the presence of the indicated stimulus. Supernatants were collected on day 1, 2 and 6 and cytokines quantified by multiplex technology. The Fold increase was calculated as: [(cytokine in stimulated culture) - (cytokine in NS culture)]/(cytokine in NS culture), where NS denotes non-stimulated cultured PBMCs. Each symbol represents data from a single subject. The data were analyzed by using the Mann-Whitney U Test, ***P<0.001, **P<0.01, *P<0.05.
(TIF)

Figure S3 **Cytokine kinetics in PBMC stimulated with CP0 protein.** PBMC from patients with chronic Chagas' disease Cardiomyopathy patients (CCC; n = 10) and non-infected individuals (NI; n = 8) were cultured in the presence of the indicated stimulus. Supernatants were collected on day 1, 2 and 6 and cytokines quantified by multiplex technology. The Fold increase was calculated as: [(cytokine in stimulated culture) - (cytokine in NS culture)]/(cytokine in NS culture), where NS denotes non-stimulated cultured PBMCs. Each symbol represents data from a single subject. The data were analyzed by using the Mann-Whitney U Test, ***P<0.001, **P<0.01, *P<0.05.
(TIF)

Figure S4 **Cytokine kinetics in PBMC stimulated with** ***T. cruzi*** **lysate.** PBMC from patients with chronic Chagas' disease Cardiomyopathy patients (CCC; n = 10) and non-infected individuals (NI; n = 8) were cultured in the presence of the indicated stimulus. Supernatants were collected on day 1, 2 and 6 and cytokines quantified by multiplex technology. The Fold increase was calculated as: [(cytokine in stimulated culture) - (cytokine in NS culture)]/(cytokine in NS culture), where NS denotes non-stimulated cultured PBMCs. Each symbol represents data from a single subject. The data were analyzed by using the Mann-Whitney U Test, ***P<0.001, **P<0.01, *P<0.05.
(TIF)

Figure S5 **Cytokine kinetics in PBMC stimulated with PHA.** PBMC from patients with chronic Chagas' disease Cardiomyopathy patients (CCC; n = 10) and non-infected individuals (NI; n = 8) were cultured in the presence of the indicated stimulus. Supernatants were collected on day 1, 2 and 6 and cytokines quantified by multiplex technology. The Fold increase was calculated as: [(cytokine in stimulated culture) - (cytokine in NS culture)]/(cytokine in NS culture), where NS denotes non-stimulated cultured PBMCs. Each symbol represents data from a single subject. The data were analyzed by using the Mann-Whitney U Test, ***P<0.001, **P<0.01, *P<0.05.
(TIF)

Figure S6 **Basal cytokine levels.** PBMC from patients with chronic Chagas' disease Cardiomyopathy patients (CCC; n = 10) and non-infected individuals (NI; n = 8) were cultured in media without any stimulation. Supernatants were collected on days 1, 2 and 6 and cytokines were quantified by multiplex technology. Each symbol represents data from a single subject. Statistical analysis was performed by using the Mann-Whitney U Test, ***P<0.001, **P<0.01, *P<0.05.
(TIF)

Figure S7 **Correlation between cytokine releases by PBMC from CCC patients upon stimulation with P2β and CP0 proteins.** Results were expressed as the sum of maximum fold increase (FI) for GM-CSF, IL-10 and TNF-α, determined as indicated in Figure 2. Spearman's correlation was performed using GraphPad Prism and data is shown (r: 0.988, *P*<0.0001).
(TIF)

Text S1 ***T. cruzi*** **lineage identification by immunophenotyping.**
(DOCX)
