# Supplementary figures and images for: Cytokine Production but Lack of Proliferation in Peripheral Blood Mononuclear Cells from Chronic Chagas' Disease Cardiomyopathy Patients in Response to T. cruzi Ribosomal P Proteins
Source: PLoS Negl Trop Dis. 2014 Jun 5;8(6):e2906. doi: 10.1371/journal.pntd.0002906 (PMC4046937; doi:10.1371/journal.pntd.0002906)

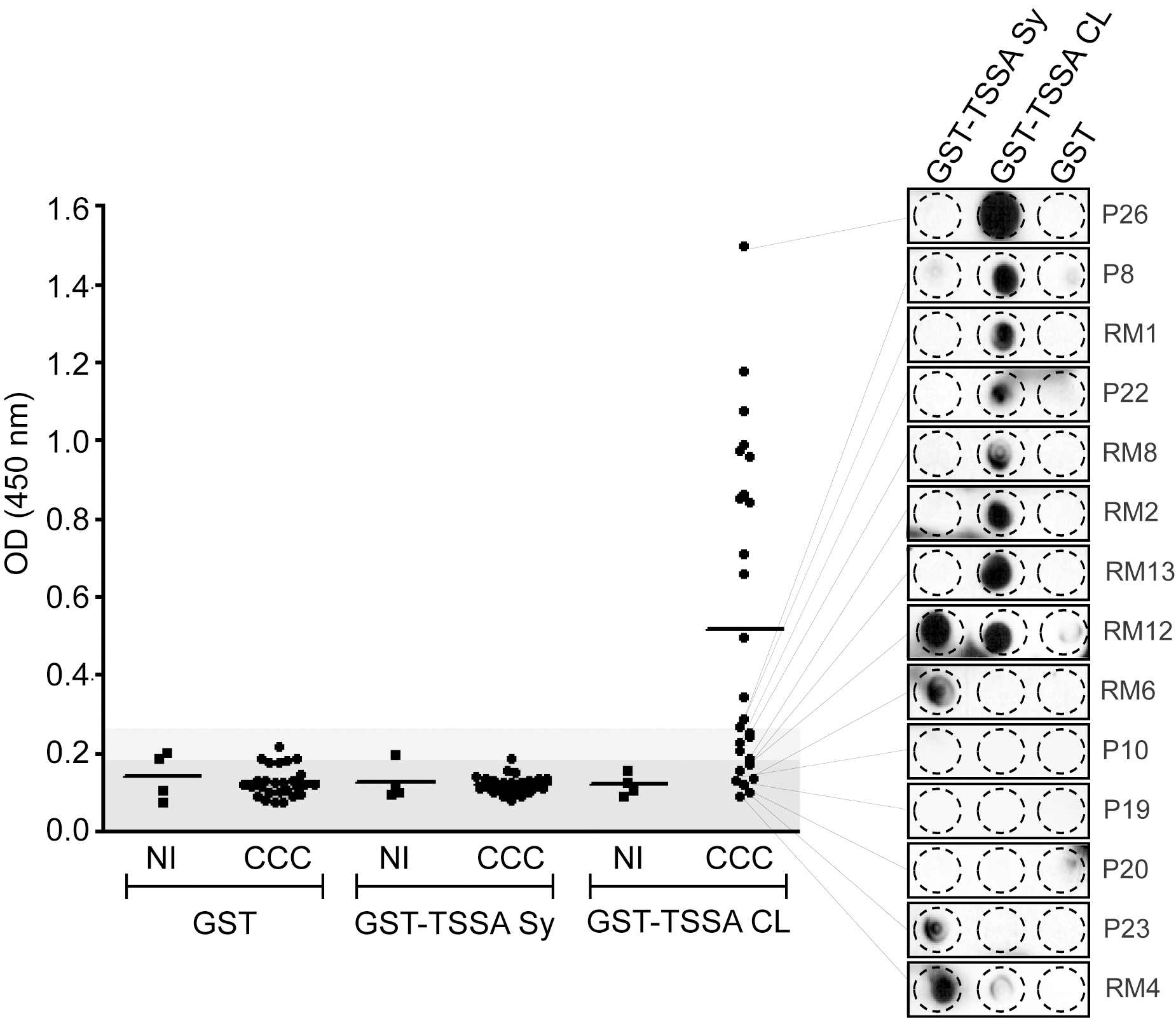

Supplement: Figure S1 — T. cruzi immunophenotyping of chagasic patients using TSSA proteins. The presence of antibodies directed against GST, GST-TSSA Sy or GST-TSSA CL proteins was determined by ELISA in chronic Chagas' disease Cardiomyopathy patients (CCC) and non-infected individuals (NI) as described under Methods. Results are expressed as means of duplicates. Negative samples were those rendering OD values below NI baseline value ±3 SD (dark grey zone). Non-conclusive samples were those rendering OD values between NI baseline value ±3 SD and NI baseline ±5 SD (light grey zone). Samples indicated to the right were re-assayed by dot-blot revealed using West-Fempto except for patient P26, which was revealed using West-Pico chemiluminescent substrate. (TIF) [file pntd.0002906.s001.tif]

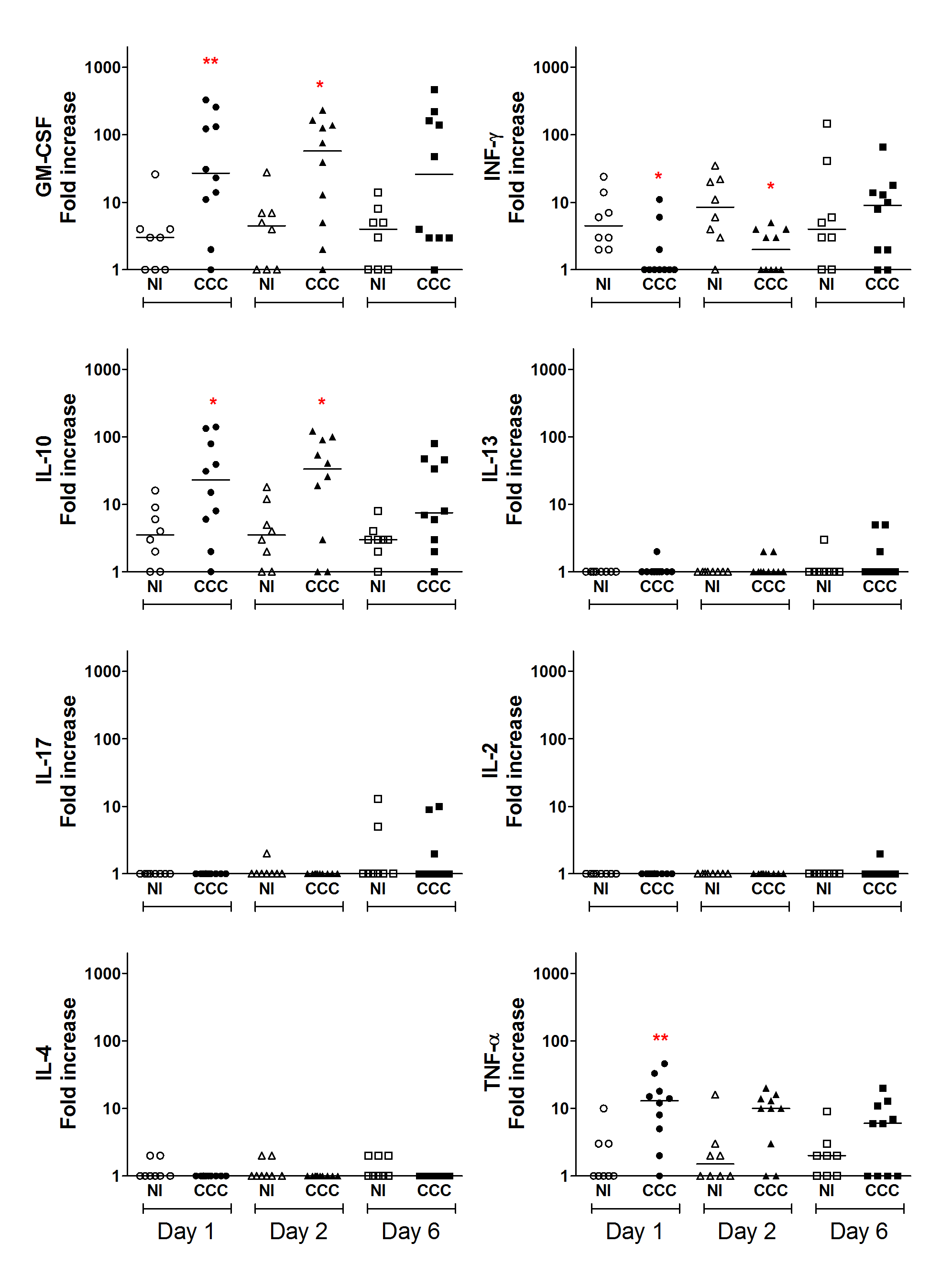

Supplement: Figure S2 — Cytokine kinetics in PBMC stimulated with P2β protein. PBMC from patients with chronic Chagas' disease Cardiomyopathy patients (CCC; n = 10) and non-infected individuals (NI; n = 8) were cultured in the presence of the indicated stimulus. Supernatants were collected on day 1, 2 and 6 and cytokines quantified by multiplex technology. The Fold increase was calculated as: [(cytokine in stimulated culture) - (cytokine in NS culture)]/(cytokine in NS culture), where NS denotes non-stimulated cultured PBMCs. Each symbol represents data from a single subject. The data were analyzed by using the Mann-Whitney U Test, ***P<0.001, **P<0.01, *P<0.05. (TIF) [file pntd.0002906.s002.tif]

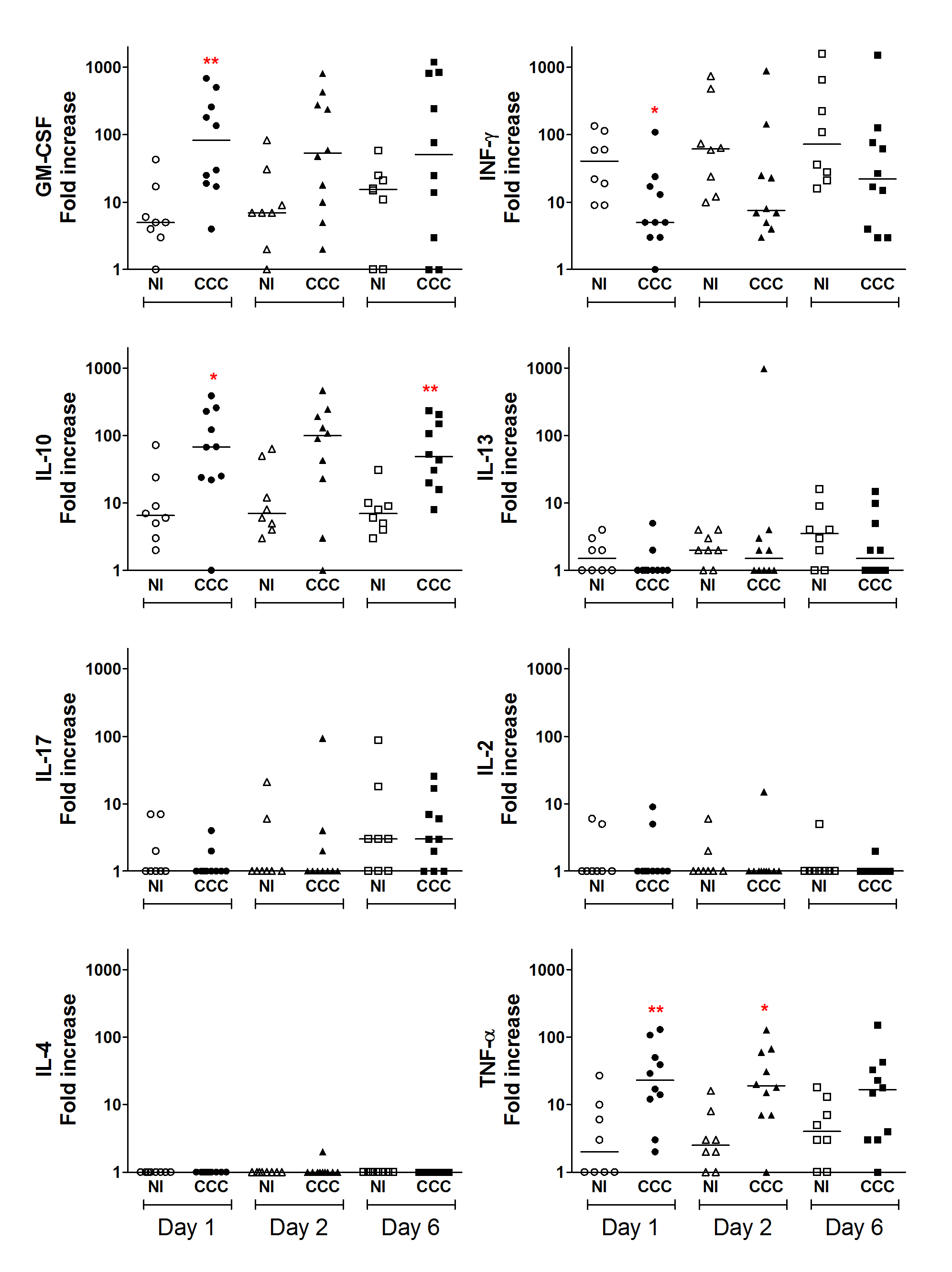

Supplement: Figure S3 — Cytokine kinetics in PBMC stimulated with CP0 protein. PBMC from patients with chronic Chagas' disease Cardiomyopathy patients (CCC; n = 10) and non-infected individuals (NI; n = 8) were cultured in the presence of the indicated stimulus. Supernatants were collected on day 1, 2 and 6 and cytokines quantified by multiplex technology. The Fold increase was calculated as: [(cytokine in stimulated culture) - (cytokine in NS culture)]/(cytokine in NS culture), where NS denotes non-stimulated cultured PBMCs. Each symbol represents data from a single subject. The data were analyzed by using the Mann-Whitney U Test, ***P<0.001, **P<0.01, *P<0.05. (TIF) [file pntd.0002906.s003.tif]

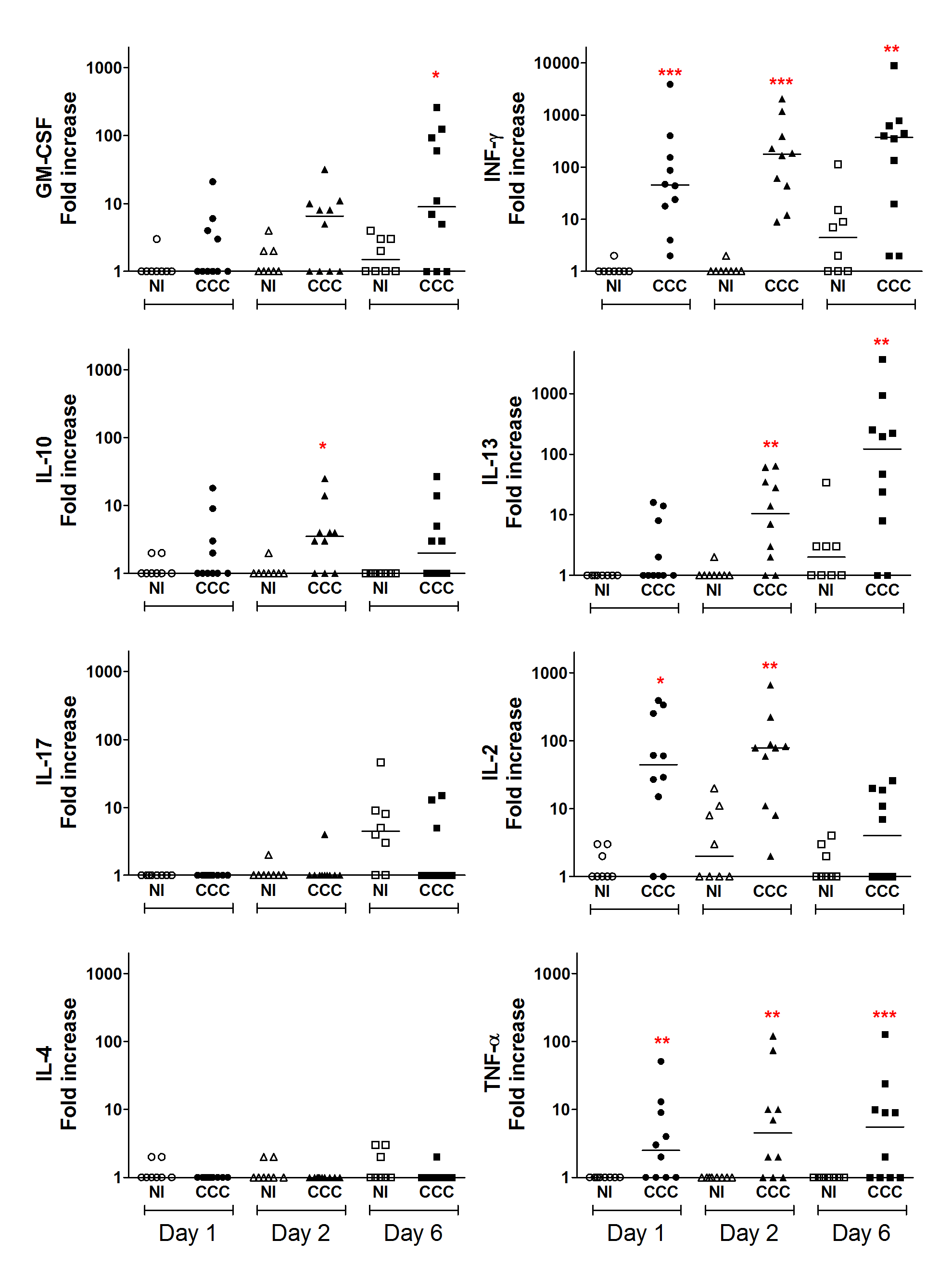

Supplement: Figure S4 — Cytokine kinetics in PBMC stimulated with T. cruzi lysate. PBMC from patients with chronic Chagas' disease Cardiomyopathy patients (CCC; n = 10) and non-infected individuals (NI; n = 8) were cultured in the presence of the indicated stimulus. Supernatants were collected on day 1, 2 and 6 and cytokines quantified by multiplex technology. The Fold increase was calculated as: [(cytokine in stimulated culture) - (cytokine in NS culture)]/(cytokine in NS culture), where NS denotes non-stimulated cultured PBMCs. Each symbol represents data from a single subject. The data were analyzed by using the Mann-Whitney U Test, ***P<0.001, **P<0.01, *P<0.05. (TIF) [file pntd.0002906.s004.tif]

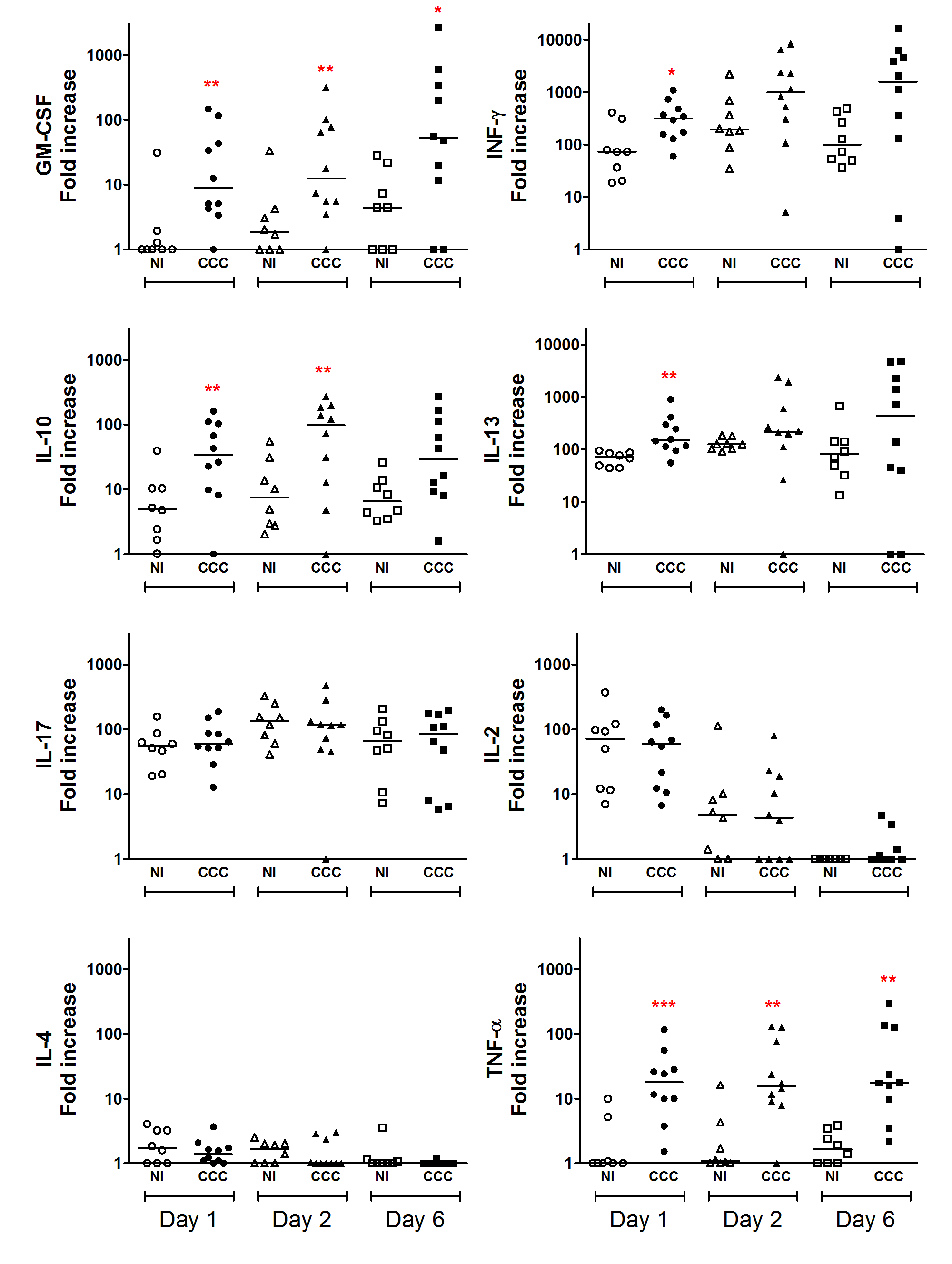

Supplement: Figure S5 — Cytokine kinetics in PBMC stimulated with PHA. PBMC from patients with chronic Chagas' disease Cardiomyopathy patients (CCC; n = 10) and non-infected individuals (NI; n = 8) were cultured in the presence of the indicated stimulus. Supernatants were collected on day 1, 2 and 6 and cytokines quantified by multiplex technology. The Fold increase was calculated as: [(cytokine in stimulated culture) - (cytokine in NS culture)]/(cytokine in NS culture), where NS denotes non-stimulated cultured PBMCs. Each symbol represents data from a single subject. The data were analyzed by using the Mann-Whitney U Test, ***P<0.001, **P<0.01, *P<0.05. (TIF) [file pntd.0002906.s005.tif]

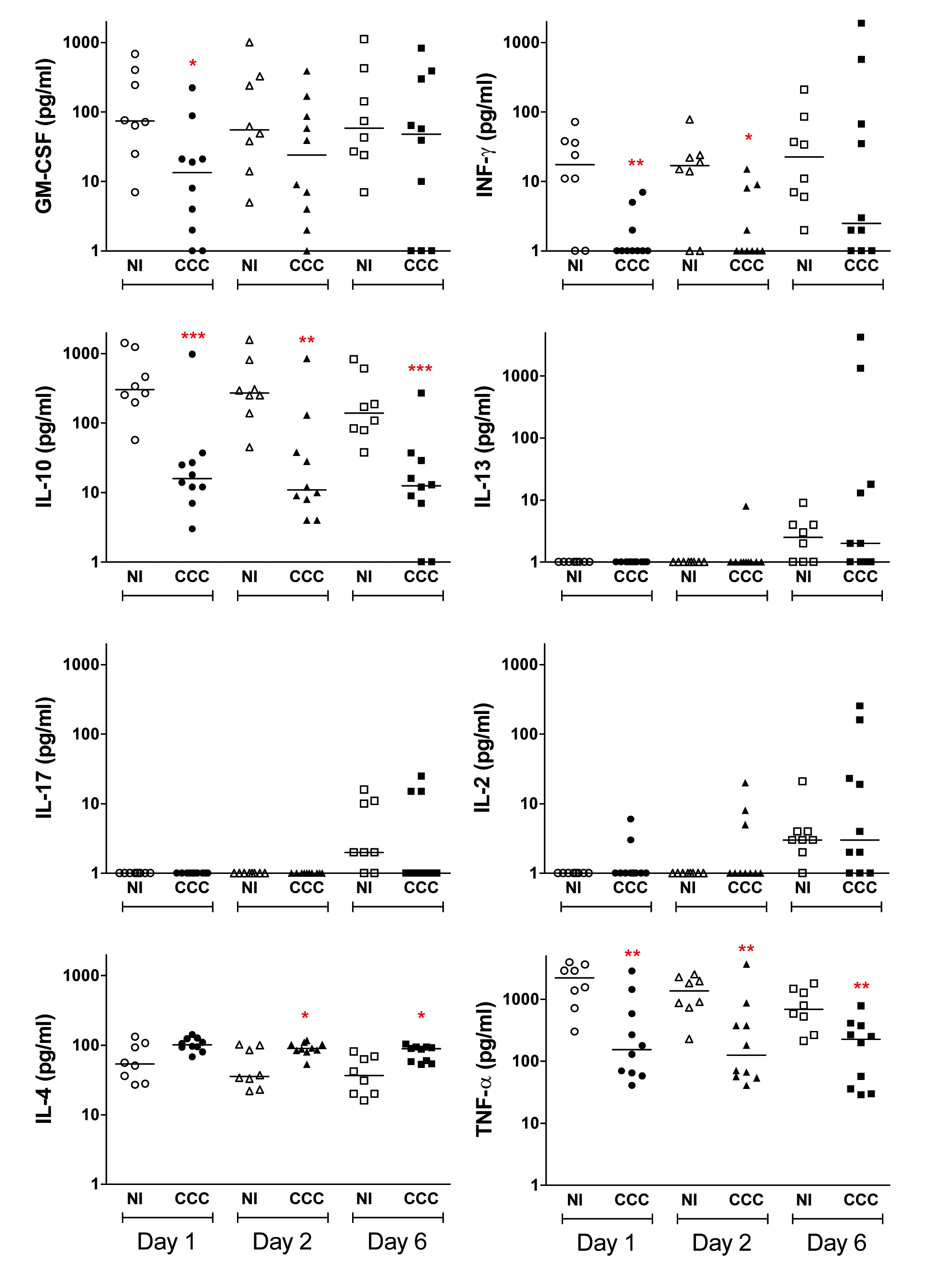

Supplement: Figure S6 — Basal cytokine levels. PBMC from patients with chronic Chagas' disease Cardiomyopathy patients (CCC; n = 10) and non-infected individuals (NI; n = 8) were cultured in media without any stimulation. Supernatants were collected on days 1, 2 and 6 and cytokines were quantified by multiplex technology. Each symbol represents data from a single subject. Statistical analysis was performed by using the Mann-Whitney U Test, ***P<0.001, **P<0.01, *P<0.05. (TIF) [file pntd.0002906.s006.tif]

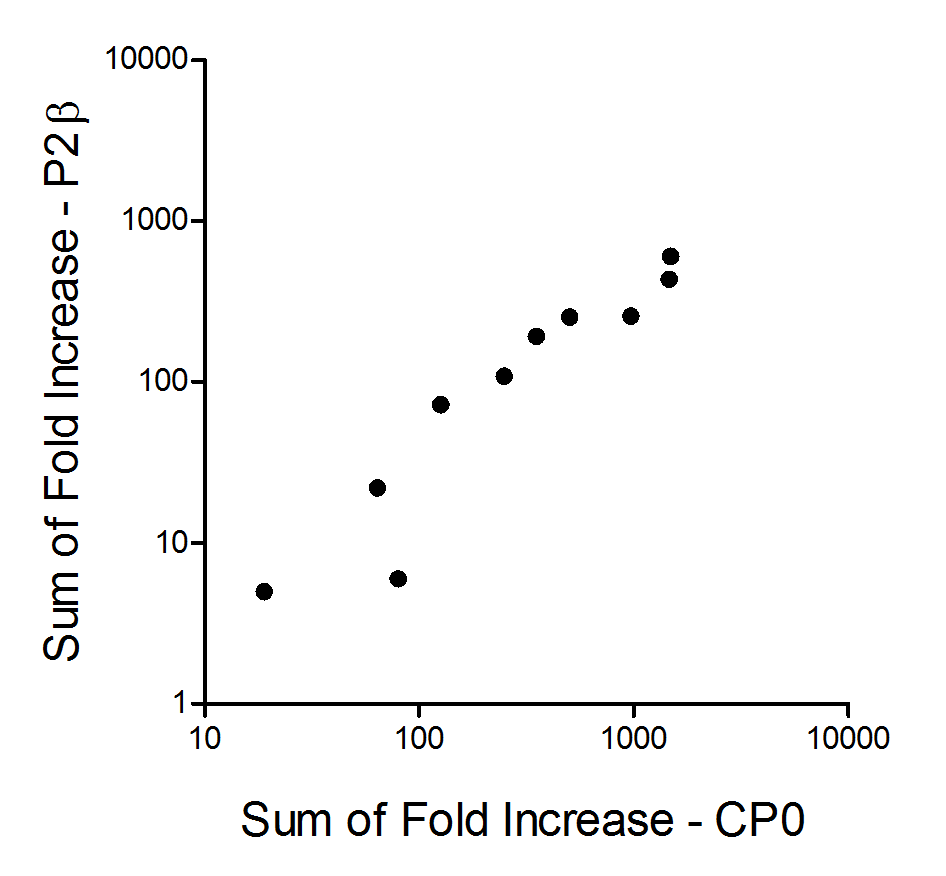

Supplement: Figure S7 — Correlation between cytokine releases by PBMC from CCC patients upon stimulation with P2β and CP0 proteins. Results were expressed as the sum of maximum fold increase (FI) for GM-CSF, IL-10 and TNF-α, determined as indicated in Figure 2. Spearman's correlation was performed using GraphPad Prism and data is shown (r: 0.988, P<0.0001). (TIF) [file pntd.0002906.s007.tif]
