# Supplementary material for: Cytokine Production but Lack of Proliferation in Peripheral Blood Mononuclear Cells from Chronic Chagas' Disease Cardiomyopathy Patients in Response to T. cruzi Ribosomal P Proteins
Source: PLoS Negl Trop Dis. 2014 Jun 5;8(6):e2906. doi: 10.1371/journal.pntd.0002906 (PMC4046937; doi:10.1371/journal.pntd.0002906)
Supplement: Text S1 — T. cruzi lineage identification by immunophenotyping. (DOCX) [file pntd.0002906.s008.docx]

**Text S1:**

***T. cruzi* lineage identification by immunophenotyping.**

In order to genotype the infecting strain(s) in our study populations, we analyzed the profile of the humoral anti-TSSA (trypomastigote small surface antigen) response. The principle of this method is based on the presence of non-cross-reactive isoforms of TSSA displayed on the surface of cell-derived trypomastigote forms from different strains [48,49], which leads to negligible cross-reactivity between them [48]. For this purpose, we firstly analyzed the reactivity of sera from CCC patients against either TSSA Sy (DTU TcI) or TSSA CL (DTUs TcII/V/VI) in conventional ELISA. The overall sensitivity of GST-TSSA CL was 74.1% (20 out of 27), though a high proportion of these samples (6 out of 20) rendered weak signals and were thus initially recorded as non-conclusive (Figure S1). None of the samples reacted against GST or GST-TSSA Sy under these conditions. To improve the sensitivity of the assay, we re-tested the samples by dot-blot, which were revealed using highly sensitive chemiluminescent methods. By doing this, we were able to detect clear-cut anti-GST-TSSA CL signals for every patient recorded as non-conclusive in our previous ELISA assays (Figure S1). Most notably, we were able to profile the anti-TSSA reactivity for 4 out of the 7 patients that yielded negative results in our ELISA assays. As shown in Figure S1, 3 out of these clearly recognized GST-TSSA Sy, 1 of them showed a mixed recognition (against both GST-TSSA CL and GST-TSSA Sy) and the remaining 3 did not react against any TSSA isoform. The antibody recognition of patient RM12 could be attributed either to a mixed *T. cruzi* infection or to an infection with strain(s) belonging to a different evolutionary group (TcIII or TcIV) which cross-recognized common epitopes in TSSA. Although these assays are not very robust and need to be improved by the addition of different DTU discriminating antigens, they provided us with a preliminary epidemiological description from our study population in the absence of direct molecular data (Table 1).
